# Phytochemical properties of *Curcuma caesia* Roxb. improve sperm quality, testosterone levels, and testicular histology in male mice

**DOI:** 10.3389/fphar.2026.1837049

**Published:** 2026-06-18

**Authors:** Ni Luh Suriani, Ting Seng Ho, Nadiah S. Alzahrani, Mohammed M. Alghamdi, Dewa Ngurah Suprapta, I. Nyoman Suarsana, Emmy Hamidah, Ni Made Rai Suarni

**Affiliations:** 1 Program Study Magister of Sustainable Finance and Development, Udayana University, Bali, Indonesia; 2 Biology Study Program, Faculty of Mathematics and Natural Sciences, Udayana University, Bali, Indonesia; 3 Back2Nature Regenerative Farm, Kuala Pilah, Malaysia; 4 College of Science, Al Baha University, Al Baha, Saudi Arabia; 5 Al Baha Health Cluster, Sub Central Blood Bank, Al Bahah, Saudi Arabia; 6 Biopesticide Laboratory, Agriculture Faculty, Udayana University, Bali, Indonesia; 7 Biochemistry Laboratory, Faculty of Veterinary Medicine, Udayana University, Bali, Indonesia; 8 Agrotechnology Study Program, Faculty of Agriculture, Universitas Islam Darul Ulum Lamongan, Lamongan, Indonesia

**Keywords:** *Curcuma caesia*, male reproductive health, oxidative stress, spermatogenesis, testosterone regulation

## Abstract

**Background:**

Medicinal plants used in traditional systems of medicine are a valuable source of bioactive compounds with therapeutic potential against oxidative stress–related disorders, including male infertility. *Curcuma caesia* Roxb. is an ethnomedicinally important species known for its antioxidant, anti-inflammatory, and aphrodisiac properties; however, its effects on male reproductive function remain insufficiently characterized.

**Purpose:**

This study aimed to evaluate the metabolites composition and dose-dependent effects of *C. caesia* extract on sperm quality, testicular histology, and testosterone levels in male mice.

**Methods:**

A total of 24 male mice were divided into four groups (n = 6) using a completely randomized design. The ethanol extract of *C. caesia* rhizomes was dissolved in distilled water and administered at designated doses: 364 mg/kg BW (P1), 728 mg/kg BW (P2), and 1,092 mg/kg BW (P3). Plant metabolites were quantified via spectrophotometry and GC-MS analysis. Serum testosterone levels were measured using ELISA, while testicular histology and morphometric parameters were assessed using hematoxylin–eosin staining and microscopic imaging.

**Results:**

Analysis revealed the presence of flavonoids, phenolics, tannins, and alkaloids. The P3 treatment significantly improved sperm concentration by 155% compared to the control. However, the P2 treatment yielded the highest percentage of viable spermatozoa (85.78%), motility (39.28%), and normal morphology (84.67%), alongside enhanced seminiferous tubule structure and spermatogenic cell density. These effects are attributed to the ROS-scavenging activity of phytochemicals, which preserve cellular morphology and support spermatogenesis. Serum testosterone levels increased in a dose-dependent manner, correlating with Leydig cell proliferation. Notably, while the moderate dose (P2) resulted in optimal reproductive outcomes, the highest dose (P3) showed a decline in sperm quality and histological alterations.

**Conclusion:**

*C. caesia* enhances male reproductive functions, supporting its traditional use as a fertility-enhancing medicinal plant. However, its effects are dose-dependent, with optimal benefits observed at moderate concentrations and potential toxicity at higher doses. These findings provide novel preclinical evidence for the use of *C. caesia* in managing male infertility and establish a pharmacological basis for future clinical investigations.

## 1 Introduction

Male reproductive health has become an increasingly critical public health concern due to the rising prevalence of infertility worldwide. Lifestyle changes, environmental exposures, and metabolic disturbances have significantly contributed to declining reproductive health in men (Gong et al., 2026). Unhealthy dietary patterns, sedentary behavior, and chronic stress are associated with various systemic disorders, including cancer, metabolic diseases, and reproductive dysfunction. Among these, male infertility represents a growing clinical challenge, estimated to contribute to nearly half of all infertility cases in couples globally (Jagadesh and Sridharan, 2026). Hormonal dysregulation, oxidative stress, environmental toxicants, and certain medications are recognized as major factors impairing male reproductive function and reducing fertility (Caroppo et al., 2026).

Oxidative stress is widely regarded as a primary pathological mechanism underlying male infertility. It arises when the production of reactive oxygen species (ROS) exceeds the capacity of endogenous antioxidant systems, resulting in cellular damage. Excessive ROS can induce lipid peroxidation of sperm membranes, mitochondrial dysfunction, DNA fragmentation, and germ cell apoptosis, ultimately leading to decreased sperm motility, viability, and fertilization capacity (Akhigbe, 2025). Furthermore, oxidative stress disrupts endocrine regulation by interfering with the hormonal signaling pathways that govern male reproductive physiology. Evidence indicates that ROS can suppress hormone production and impair communication within the reproductive endocrine axis (Hussain et al., 2023).

Male reproductive function is controlled by the hypothalamic–pituitary–gonadal (HPG) axis, a complex endocrine network responsible for coordinating hormone secretion and spermatogenesis. In this axis, the hypothalamus releases gonadotropin-releasing hormone (GnRH), which stimulates the anterior pituitary gland to produce luteinizing hormone (LH) and follicle-stimulating hormone (FSH). LH acts primarily on Leydig cells in the testes to stimulate testosterone synthesis, while FSH regulates Sertoli cell function and supports spermatogenesis. Disruption of this signaling cascade can impair testosterone production and compromise sperm development. Oxidative stress has been shown to interfere with multiple components of the HPG axis by reducing the secretion of GnRH, LH, and FSH and inhibiting steroidogenic enzyme activity (Hussain et al., 2023).

Within the testes, Leydig cells play a central role in steroidogenesis. These specialized interstitial cells synthesize testosterone, the primary androgen responsible for maintaining spermatogenesis, supporting secondary sexual characteristics, and regulating reproductive behavior. However, Leydig cells are highly sensitive to oxidative stress due to their intense mitochondrial activity. Elevated ROS levels can damage mitochondrial membranes, inhibit enzymes such as steroidogenic acute regulatory (StAR) protein, and trigger apoptosis in Leydig cells, resulting in decreased testosterone production and endocrine imbalance (Yang et al., 2025). Experimental studies have demonstrated that oxidative stress-induced apoptosis in Leydig cells directly impairs testosterone biosynthesis, contributing to male infertility (Jagadesh and Sridharan, 2026).

Since oxidative stress plays a major role in male reproductive failure, many studies have focused on therapeutic approaches that bolster antioxidant defenses (Wang et al., 2025). Due to their capacity to neutralize free radicals and restore redox balance, natural antioxidants derived from medicinal plants have drawn significant attention (Tarlan et al., 2026). Phytochemicals—including flavonoids, phenolics, tannins, and alkaloids—possess strong antioxidant and anti-inflammatory qualities that may shield endocrine tissues and spermatogenic cells from oxidative damage. In experimental models, plant-derived antioxidants have been shown to enhance sperm quality, increase testosterone levels, and restore testicular structure (Ahmad and Ahmad, 2025). These protective actions preserve the integrity of Leydig cells and the activity of steroidogenic enzymes (Bouhadana et al., 2025), resulting in enhanced sperm motility, concentration, and morphology (Nouri et al., 2026).

*Curcuma caesia* Roxb., a rhizomatous botanical drug belonging to the Zingiberaceae family, has gained growing interest for its pharmacological potential (Suriani et al., 2026). It is widely used in traditional medicine across South and Southeast Asia to treat inflammatory disorders, metabolic diseases, and reproductive ailments. Phytochemical investigations have revealed that the rhizome contains diverse bioactive metabolites, including phenolics, flavonoids, terpenoids, and essential oils such as camphor, 1,8-cineole, and ar-turmerone. These metabolites exhibit strong antioxidant, antimicrobial, and anti-inflammatory activities (Leela and Adheeba, 2024).

Recent pharmacological studies have further demonstrated that *C. caesia* possesses a high antioxidant capacity associated with abundant phenolic metabolites (Rajwade and Kulkarni, 2025). Through these mechanisms, *C. caesia* may protect reproductive cells from ROS-induced damage and support normal testicular function. Despite these promising properties, few studies have examined its role in regulating male reproductive endocrine function, particularly its effects on sperm quality, testosterone production, and testicular histological integrity.

Based on the link between oxidative stress and endocrine regulation, this study hypothesizes that phytochemical-rich extracts of *C. caesia* can improve male reproductive function by enhancing antioxidant defenses. Antioxidant metabolites in *C. caesia* may protect Leydig and spermatogenic cells, preserve steroidogenic enzyme activity, and support testosterone biosynthesis within the HPG axis (Yang et al., 2025). Therefore, this study aims to determine the optimal dosage of *C. caesia* in relation to its phytochemical activity and to evaluate its effects on sperm quality, testicular histology, and testosterone levels in male mice. These findings are expected to demonstrate a dose-dependent relationship between antioxidant activity and reproductive endocrine responses, providing mechanistic insight into *C. caesia* as a natural therapeutic candidate for improving male reproductive health.

## 2 Materials and methods

### 2.1 Study location and duration

The study was conducted between April 2025 and February 2026 at several research facilities. Animal experiments and histological analyses were performed at the Veterinary Laboratory and the Biopesticide Laboratory of Udayana University, Bali, Indonesia. Phytochemical and antioxidant analyses were carried out at the Laboratory of the Faculty of Mathematics and Natural Sciences, Udayana University, while preparation of *C. caesia* powder was performed at the Back2Nature Laboratory, Kuala Pilah, Malaysia.

### 2.2 Experimental design

The experiment was conducted using a completely randomized design (CRD) consisting of four treatment groups with six replicates each. A total of 24 male mice were randomly assigned to the following groups:

P0: Control group (distilled water)
P1: *C*. *caesia* extract at 364 mg/kg body weight
P2: *C*. *caesia* extract at 728 mg/kg body weight
P3: *C*. *caesia* extract at 1,092 mg/kg body weight

### 2.3 Experimental animals and husbandry

A total of 24 adult male mice weighing 25–33 g (average approximately 25 g) were used in this study. The animals were obtained from a licensed animal supplier in Denpasar, Bali, Indonesia. Prior to experimentation, the mice were acclimatized for 1 week under controlled laboratory conditions.

The animals were housed in four cages, with six mice per cage, under standard environmental conditions with controlled temperature, humidity, and a 12 h light/12 h dark cycle. Standard laboratory feed and drinking water were provided *ad libitum* in accordance with established laboratory animal care guidelines (Albus, 2012). Mice in the cage treatment are ensured that there is no pressure and no one is injured, control is carried out on the rats in the cage every day.

### 2.4 Collection, authorization, and harbarium deposition

*Curcuma caesia* rhizomes were collected from Back2Nature Regenerative Farm, Kuala Pilah, Negeri Sembilan, Malaysia. *C. caesia* has been identified at Forest Research Institute Malaysia (FRIM), Malaysia and there is a genetic scanning, stored at the FRIM Malaysia botanical drugarium with sample ID number 005826 (Supplementary Data Sheet 1).

### 2.5 Preparation of *Curcuma caesia* samples

For the extraction purpose, 4 kg rhizomes of *C. caesia* were collected, cut it into small pieces, air-dried for 5 days, and blended to form a powder. A 1,000 g powder of rhizomes of *C. caesia* was separately dissolved in 10 L of 70% ethanol with a weight/volume ratio of 1:10 and kept for 48 h at 28 °C. After that was filtered by used paper No. 1. and ethanol was evaporated within a rotary evaporator (Iwaki, Tokyo) at 40 °C (Suriani et al., 2020). The result of the evaporator is an extract that is ready to treatment to the mice and for analysis of phytochemical of *C. caesia* rhizomes. For treatment preparation, the extract was dissolved in distilled water according to the designated experimental doses: 364 mg/kg BW, 728 mg/kg BW, and 1,092 mg/kg BW (Tsao et al., 2022).

### 2.6 Administration of *Curcuma caesia*

*Curcuma caesia* extract was dissolved in distilled water (vortex) and administered orally to the experimental animalsaccording to dosis P0: Control group (distilled water), P1: *C. caesia* extract at 364 mg/kg body weight, P2: *C. caesia* extract at 728 mg/kg body weight, P3: *C. caesia* extract at 1,092 mg/kg body weight. The prepared *C*. *caesia* solution was administered orally to the mice once daily in the morning using a gavage technique. Each mouse received 0.5 mL of the prepared solution, corresponding to the designated treatment dose. The treatment period lasted 35 consecutive days (Silalahi et al., 2023).

### 2.7 Sample collection

On day 36, the mice were anesthetized for euthanasia. Ketamine 40 mg/kg body weight and zylazine 5 mg/kg body weight were injected intramuscularly into the thigh muscle of mice. And after the anesthesia stage is further carried out, cervical dislocation follows established laboratory procedures (AVMA, 2020). Animals that have been euthanized are then incinerated (for destruction). The testes and epididymides were carefully removed and rinsed with 0.9% NaCl solution to remove blood and debris. The cauda epididymis was minced in 1 mL of 0.9% NaCl solution to obtain a sperm suspension for sperm quality analysis. Testicular tissues were preserved for histological examination, and blood samples were collected via cardiac puncture for serum testosterone analysis according to the guidelines established by the World Health Organization (WHO, 2010).

### 2.8 Sperm quality assessment

Sperm quality analysis was conducted using a Zeiss Primo Star 3 light microscope equipped with an Axiocam 208 color camera at the Biopesticide Laboratory of Udayana University. Following sacrifice, the right epididymis was excised and processed to obtain a sperm suspension. Sperm concentration was determined using a hemocytometer, following the method described by Greco et al. (2021). Additional parameters, including sperm motility, morphology, and vitality, were assessed using Eosin-Nigrosin staining according to the guidelines established by the World Health Organization (WHO, 2010).

### 2.9 Testicular histology

Testicular tissues were processed for histological analysis using the paraffin embedding technique followed by hematoxylin–eosin (H&E) staining at the Veterinary Laboratory of Udayana University. Histological sections were examined under a light microscope to evaluate seminiferous tubule structure and spermatogenic cell organization (Wahyuni et al., 2023).

### 2.10 Preparation of plant extract for phytochemicals and GC-MS analysis

*Curcuma caesia* powder was extracted using ethanol to obtain a concentrated plant extract for phytochemical and antioxidant analysis. The extract was concentrated using a rotary evaporator before further chemical analyses. The extract was subsequently analyzed to determine total phenolic, flavonoid, tannin, alkaloid, and antioxidant contents, and using GC-MS to obtain more detailed metabolites(s) (Sharma et al., 2020).

#### 2.10.1 Determination of total phenolic content

Total phenolic content was determined using the Folin–Ciocalteu colorimetric method with gallic acid as the standard. A calibration curve was prepared using gallic acid concentrations of 10, 20, 30, and 50 ppm. The reaction mixture consisted of 0.4 mL Folin–Ciocalteu reagent and 4.0 mL of 7% Na_2_CO_3_ solution, followed by incubation at room temperature for 2 h. Absorbance was measured at 744.8 nm using a UV–Vis spectrophotometer. Results were expressed as milligrams of gallic acid equivalents (GAE) (Sun et al., 2024; Tang et al., 2024).

#### 2.10.2 Determination of total flavonoid content

Total flavonoid content was measured using the aluminum chloride colorimetric method with quercetin as the standard. Standard solutions were prepared at concentrations of 10–50 ppm. The reaction mixture consisted of quercetin solution, 10% AlCl_3_, potassium acetate, and distilled water, followed by incubation at room temperature for 30 min. Absorbance was measured at 431 nm using a UV–Vis spectrophotometer. Results were expressed as quercetin equivalents (QE) (Mart et al., 2022; Tanesib et al., 2023).

#### 2.10.3 Determination of tannin content

Tannin content was determined spectrophotometrically using the Folin–Denis method with tannic acid as the standard. The sample extract was treated with Folin–Denis reagent and 5% Na_2_CO_3_, followed by incubation for 30 min. Absorbance was measured at 725 nm using a spectrophotometer (Nguyen and Chuyen, 2020).

#### 2.10.4 Determination of alkaloid content

Alkaloid content was determined using a spectrophotometric method. The extract was dissolved in ethanol and treated with 2 N HCl, followed by extraction with chloroform. After further reaction with phosphate buffer (pH 4.7) and bromocresol green (BCG) solution, the absorbance of the chloroform phase was measured at 273 nm, and mg caffeine equivalents (mg CE/g) of extract are used to express the results (Liberal et al., 2024).

### 2.11 Antioxidant capacity

Creation of a gallic acid standard curve at different concentrations (0–2 mg/L). 0.05 g of the sample were weighed, diluted with 99.9% methanol to a volume of 5 mL in a volumetric flask, vortexed, and centrifuged for 15 min at 3,000 rpm. After pipetting 0.5 of the standard and supernatant into the test tube, 0.5 mL of 0.1 mM DPPH (in 99.9% methanol solvent) was added, and the mixture was vortexed. The absorbance was then measured at λ 517 nm after it was incubated at 25 °C for 30 min to provide DPPH time to react with hydrogen atoms donated by the sample antioxidant. The linear regression equation formula y = ax + b was used to determine the antioxidant capacity (Flieger et al., 2021).

### 2.12 Gas chromatography–mass spectrometry (GC-MS) analysis

*Curcuma caesia r*hizomes extracts’ bioactive components were identified using gas chromatography–mass spectrometry (GC–MS; QP2010SE, Shimadzu, Japan). By comparing mass spectra with those in the GC–MS library database, metabolites(s) were identified. The Joint Mathematics and Natural Sciences Laboratory at Udayana University was the site of the analyses (Suriani et al., 2026).

### 2.13 Statistical analysis

All experimental data were expressed as mean ± standard deviation (SD). Statistical analysis was performed using one-way analysis of variance (ANOVA) to determine significant differences among treatment groups (Montgomery, 2020). When significant differences were detected, post-hoc multiple comparison tests using Tukey’s honestly significant difference (HSD) test were conducted to compare individual group means (Tukey, 1949). Statistical analyses were performed using SPSS software (version 26.0; IBM Corp., Armonk, NY, United States), following standard procedures for biological data analysis (Field, 2018). A p-value < 0.05 was considered statistically significant. Graphs and data visualization were generated using GraphPad Prism software (version 9.0).

## 3 Results

### 3.1 Phytochemical and GC-MS analysis of *Curcuma caesia*

Phytochemical analysis revealed that *C. caesia* contained substantial levels of bioactive metabolites(s), including flavonoids, phenolic metabolites(s), tannins, and alkaloids, along with strong antioxidant capacity (Table 1). The total flavonoid content was 75.32 ± 0.63 mg QE/100 mL, while total phenolic content reached 389.56 ± 0.81 mg GAE/100 g. The tannin content was 154.48 ± 0.36 mg TAE/100 g, and alkaloid content was 0.45% ± 0.42%. In addition, the antioxidant capacity of *C. caesia* extract was 897.21 ± 0.76 ppm GAE, indicating a very high antioxidant potential. These findings suggest that *C. caesia* is rich in phytochemicals capable of contributing to antioxidant activity and potential biological effects. 25 phytochemical substances, mostly from sesquiterpenoid class, were found in the *C. caesia* extract using GC-MS analysis (Table 2). With the highest relative concentration of 24.34% δ-elemene was the main chemical. It was followed by camphor (10.57%), germacrene b (10.09%), valencene (7.89%), curzerene (6.95%), and α-humulene (6.86%).

**TABLE 1:** Results of phytochemical analysis of *Curcuma caesia*.

Parameter	Methods Used	Unit	Samples
Total Flavonoid	Spectrophotometry (UV Vis)	mg QE/100 mL	75.32 ± 0.63
Total Phenol	Spectrophotometry (UV Vis)	mgGAE/100 mL	389.56 ± 0.81
Tannin	Spectrophotometry (UV Vis)	mgTAE/100 g	154.48 ± 0.36
Antioxidant capacity	Spectrophotometry (UV Vis)	ppm GAE	897.21 ± 0.76
Alkaloid	Spectrophotometry (UV Vis)	%	0.45 ± 0.42

Values are the average of triplicates. Different superscript letters within the same column indicate statistically significant differences (p < 0.05) using DMRT following ANOVA.

**TABLE 2:** Phytochemical metabolites(s) of *Curcuma caesia* by GC-MS Analysis.

No	Ret.Time	Area	Conc (%)	Metabolites(s) name	Class	Reported biological activities	References
1	5.26	106,798,956	10.57	Campor	Monoterpenoid	Anti-inflammatory, analgesic, improves blood circulation	Shabbir et al. (2026)
2	5.476	15,914,492	1.57	Isoborneol	Monoterpenoid	Antimicrobial, anti-inflammatory	Andrade (2013)
3	7.317	4,793,040	0.47	Tridecane	Alkana	No significant biological activity	–
4	7.886	27,389,193	2.71	δ-Elemene	Sesquiterpenoid	Anti-inflammatory, anticancer	Ribeiro et al. (2026)
5	8.556	17,343,103	1.72	δ-Elemene	Sesquiterpenoid	Anti-inflammatory, anticancer	Ribeiro et al. (2026)
6	8.658	246,067,217	24.34	δ-Elemene	Sesquiterpenoid	Strong anti-inflammatory, tissue protective	Ribeiro et al. (2026)
7	9.091	79,719,645	7.89	Valencene	Sesquiterpenoid	Anti-inflammatory, antioxidant	Liu (2024)
8	9.186	101,982,940	10.09	Germacreno B	Sesquiterpenoid	Anti-inflammatory, antimicrobial	Luu-dam et al. (2023)
9	9.36	1,338,608	0.13	Hexaborane 12	Boron compound	No relevant biological activity	–
10	9.415	38,290,098	3.79	Cyclobutane derivative	Terpenoid	Potential anti-inflammatory activity	Malel, (2026)
11	9.502	15,243,600	1.51	Azulene derivative	Sesquiterpenoid	Anti-inflammatory	Beata and Goslinski (2021)
12	9.555	6,292,100	0.62	Germacrene D	Sesquiterpenoid	Anti-inflammatory, antimicrobial	Luu-dam et al. (2023)
13	9.614	69,360,727	6.86	α-Humulene	Sesquiterpenoid	Strong anti-inflammatory, antioxidant	Beata and Goslinski (2021)
14	9.938	43,796,401	4.33	Cyclodecadiene derivative	Sesquiterpenoid	Anti-inflammatory	Orellana-paucar (2024)
15	10.066	70,266,652	6.95	Curzerene	Sesquiterpenoid	Anti-inflammatory, antimicrobial, antioxidant, anti cancer	Shabbir et al. (2026)
16	10.24	1,615,076	0.16	Hexaborane-12	Boron compound	No significant activity	–
17	10.265	9,949,639	0.98	Azulene derivative	Sesquiterpenoid	Anti-inflammatory	Chen et al. (2023)
18	10.374	12,468,211	1.23	Bicyclic terpene	Terpenoid	Anti-inflammatory	Almeida et al. (2025)
19	10.574	5,458,049	0.54	Cyclopropea zulene	Sesquiterpenoid	Anti-inflammatory	Chen et al. (2023)
20	10.664	17,174,236	1.7	Germacrene B	Sesquiterpenoid	Anti-inflammatory	Luu-dam et al. (2023)
21	10.722	14,912,296	1.48	Neoisolongifolene	Sesquiterpenoid	Anti-inflammatory, antimicrobial	Malel, (2026)
22	10.76	9,625,864	0.95	Benzopyran derivative	Flavonoid-like	Antioxidant	Chen et al. (2023)
23	10.82	4,433,518	0.44	Cyclopropanap hthalene	Terpenoid	Potential anti-inflammatory	Malel, (2026)
24	10.974	66,226,784	6.55	Germacrene B	Sesquiterpenoid	Anti-inflammatory	Luu-dam et al. (2023)
25	11.196	24,392,244	2.41	Pentanediol derivative	Ester	Low biological activity	–

### 3.2 Effect of *Curcuma caesia* on sperm concentration

**FIGURE 1:**
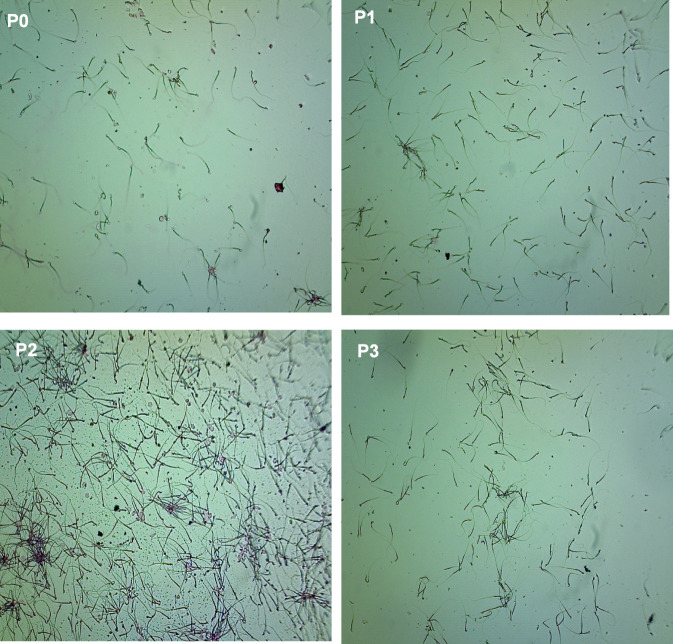
Spermatozoa concentration observed under microscope (100×) following various treatments; P0 - Control, P1- *C caesia* powder at 364 mg/kg body weight, P2-*Curcuma caesia* powder at 728 mg/kg body weight, and P3-*C caesia* powder at 1,092 mg/kg body weight.

Administration of *C. caesia* significantly affected sperm concentration in male mice (Table 3). The highest sperm concentration was observed in the 728 mg/kg body weight (P2) treatment group, reaching 47.17 × 10^6^/mL, followed by the 364 mg/kg BW (P1) group. Although the 1,092 mg/kg BW (P3) treatment produced a higher sperm concentration than the control group, it showed a decline compared with the P1 and P2 treatments. The control group (P0) exhibited the lowest sperm concentration (18.50 × 10^6^/mL). Microscopic observations supported these findings, showing that sperm density was highest in P2, followed by P1, P3, and the control group (Figure 1). These results indicate that moderate doses of *C. caesia* produce the most pronounced improvement in sperm production.

**TABLE 3:** Sperm concentration in mice treated with *Curcuma caesia*.

Treatment	Spermatozoa concentration (10^6^/mL)	Percentage increase vs. control (%)
Control (P0)	18.50 ± 4.04^a^	-
364 mg/kgBW (P1)	43.50 ± 7.66^c^	135.14
728 mg/kgBW (P2)	47.17 ± 2.23^c^	155.00
1.092 mg/kgBW (P3)	30.67 ± 7.10^b^	65.78

Values are the average of six replicates. Different superscript letters within the same column indicate statistically significant differences (p < 0.05) using DMRT following ANOVA.

### 3.3 Sperm viability

**FIGURE 2:**
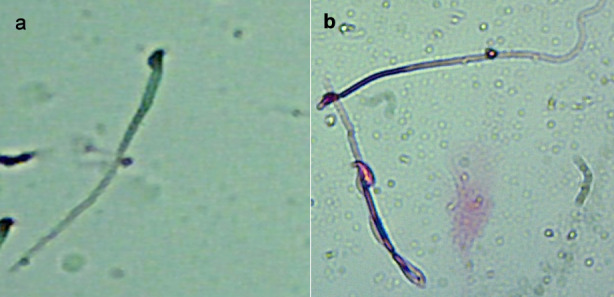
Spermatozoa normal **(a)** and abnormal **(b)** under microscope (400×).

Significant differences in sperm viability were observed between the control and treatment groups (Table 4). The highest percentage of viable spermatozoa (85.78%) was observed in the 728 mg/kg BW (P2) treatment group. Increasing the dose to 1,092 mg/kg BW (P3) resulted in a marked decrease in sperm viability (44.89%). Correspondingly, the lowest percentage of non-viable spermatozoa (14.22%) was observed in the P2 group, whereas the control group exhibited the highest proportion of non-viable sperm (Figure 2).

**TABLE 4:** Average viability of spermatozoa.

Treatment	Viable (%)	Non-viable (%)
Control (P0)	28.39 ± 3.87^a^	71.61 ± 3.87^d^
364 mg/kgBW (P1)	74.06 ± 12.08^c^	25.94 ± 12.08^b^
728 mg/kgBW (P2)	85.78 ± 3.13^d^	14.22 ± 3.13^a^
1.092 mg/kgBW (P3)	44.89 ± 9.96^b^	55.11 ± 9.96^c^

Values are the average of six replicates. Different superscript letters within the same column indicate statistically significant differences (p <0.05) using DMRT following ANOVA.

### 3.4 Sperm morphology

Sperm morphology analysis also revealed significant differences among treatments (Table 5). The highest proportion of normal spermatozoa (84.67%) was observed in the P2 treatment (728 mg/kg BW) (Figure 2a). In contrast, the control group exhibited the lowest percentage of normal spermatozoa (27.72%) (Figure 2b). At the highest treatment dose (1,092 mg/kg BW), the proportion of normal spermatozoa decreased substantially, while abnormal sperm increased to 60.50%, suggesting a possible adverse effect at excessive doses.

**TABLE 5:** Average morphology of spermatozoa.

Treatment	Normal (%)	Abnormal (%)
Control (P0)	27.72 ± 5.37^a^	72.28 ± 5.37^c^
364 mg/kgBW (P1)	76.28 ± 12.40^c^	23.72 ± 12.40^a^
728 mg/kgBW (P2)	84.67 ± 4.80^c^	15.33 ± 4.88^a^
1.092 mg/kgBW (P3)	39.56 ± 10.12^b^	60.50 ± 10.19^b^

Values are average of six replicates. Different superscript letters within the same column indicate statistically significant differences (p <0.05) using DMRT following ANOVA.

### 3.5 Sperm motility

Sperm motility was significantly improved in mice treated with *C. caesia* (Table 6). The highest motility percentage (39.28%) was observed in the 728 mg/kg BW group (P2), followed by P1 (34.63%). However, motility declined at the highest dose (P3 = 22.67%). The control group exhibited the lowest motility and the highest proportion of non-motile spermatozoa.

**TABLE 6:** Average spermatozoa motility.

Treatment	Motile (%)	Non-motile (%)
Control	19.89 ± 3.54^a^	40.17 ± 7.68^c^
364 mg/kgBW	34.63 ± 8.65^b^	15.00 ± 4.65^a^
728 mg/kgBW	39.28 ± 5.27^b^	13.67 ± 3.50^a^
1.092 mg/kgBW	22.67 ± 1.75^a^	29.50 ± 4.93^b^

Values are the average of six replicates. Different superscript letters within the same column indicate statistically significant differences (p <0.05) using DMRT following ANOVA.

### 3.6 Effect of *Curcuma caesia* on testicular histology in mice

**FIGURE 3:**
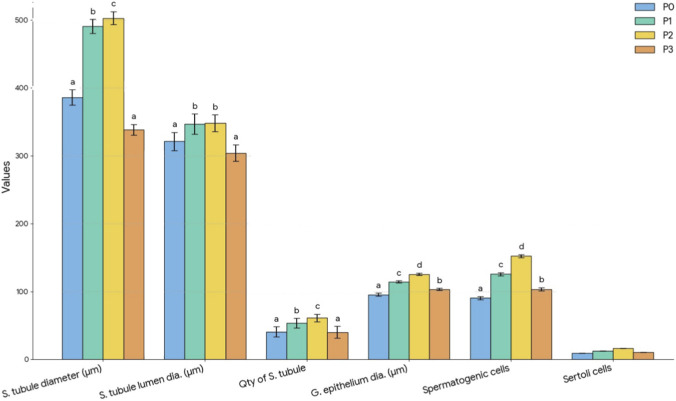
Testicular histomorphometrical parameters across different groups of mice treated with *Curcuma caesia* water extract Values are the average of six replicates. Different superscript letters within the same column indicate statistically significant differences (p < 0.05) using DMRT following ANOVA.

**FIGURE 4:**
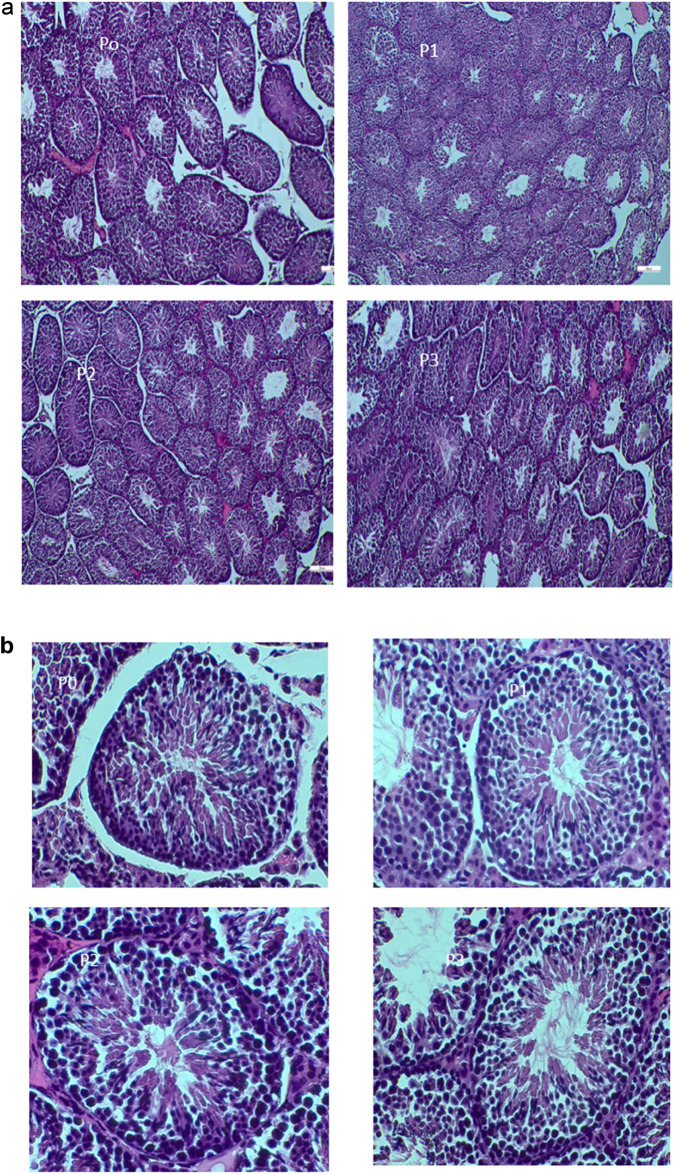
**(a)** Testicular histomorphometrical in mice treated with *Curcuma caesia* extract under microscope 100× following various treatments: P0 - Control, P1*- Curcuma caesia* powder at 364 mg/kg body weight, P2- *Curcuma caesia* powder at 728 mg/kg body weight, and P3-*Curcuma caesia* powder at 1,092 mg/kg body weight. **(b)** Testicular histomorphometrical in mice treated with *Curcuma caesia* extract under microscope 400× following various treatments; P0 - Control, P1- *Curcuma caesia* powder at 364 mg/kg body weight, P2-*Curcuma caesia* powder at 728 mg/kg body weight, and P3- *Curcuma caesia* powder at 1,092 mg/kg body weight.

Histological examination demonstrated that *C. caesia* significantly influenced testicular histomorphometry (Figures 3, 4). The 728 mg/kg BW (P2) treatment produced the most pronounced improvements in testicular structure, including increased seminiferous tubule diameter, increased lumen diameter, increased number of seminiferous tubules, thicker germinal epithelium, and increased spermatogenic cell numbers. At the highest dose (1,092 mg/kg BW), most histological parameters declined, and the seminiferous tubules appeared less compact with relatively empty lumens.

### 3.7 Effect of *Curcuma caesia* on the testosterone hormone of mice

Serum testosterone levels differed significantly among treatments (Table 7). The highest testosterone concentration was observed in the 1,092 mg/kg BW (P3) group (13.344 ng/mL), followed by the 728 mg/kg BW (P2) group. Although the 364 mg/kg BW (P1) treatment showed an increase compared with the control group, the difference was not statistically significant.

**TABLE 7:** Testosterone hormone of mice.

Treatment	Testosterone (ng/mL)
Control (P0)	8.051 ± 1.850^a^
364 mg/kgBW (P1)	9.763 ± 1.480^a^
728 mg/kgBW (P2)	10.004 ± 2.307^ab^
1.092 mg/kgBW (P3)	13.344 ± 2.738^b^

Values are the average of six replicates. Different superscript letters within the same column indicate statistically significant differences (p < 0.05) using DMRT following ANOVA.

### 3.8 Relationship between leydig cells and testosterone levels

**FIGURE 5:**
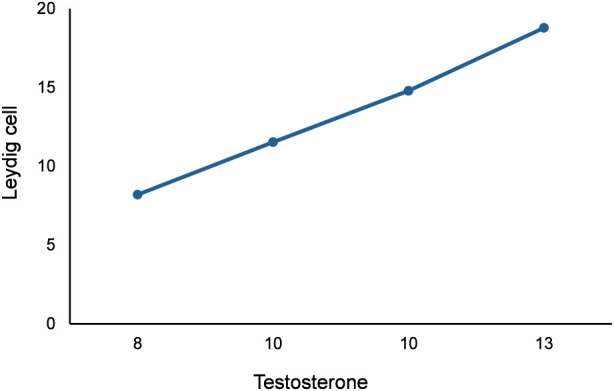
Relationship between Leydig cell and testosterone hormone in mice treated with *Curcuma caesia*.

A positive correlation was observed between the number of Leydig cells (Leydig cell counts were determined by counting the number of cells in five randomly selected fields of view under ×400 magnification) and serum testosterone levels (Figure 5; Table 8). Treatments that increased the number of Leydig cells were associated with elevated testosterone concentrations. The highest Leydig cell counts and testosterone levels were observed in the 1,092 mg/kg BW treatment, indicating that Leydig cell proliferation may contribute to enhanced androgen production in response to *C. caesia* administration.

**TABLE 8:** Leydig cell testicular in mice treated with *Curcuma caesia* extract.

Treatment	Leydig cell
Control (P0)	8.20 ± 1.23^a^
364 mg/kgW (P1)	11.53 ± 1.18^a^
728 mg/kgW (P2)	14.79 ± 2.21^ab^
1.092 mg/kgW (P3)	18.78 ± 2.41^b^

Values are average of six replicates. Different superscript letters within the same column indicate statistically significant differences (p < 0.05) using DMRT following ANOVA.

## 4 Discussion

The present study demonstrates that the administration of *C. caesia* ethanol extract significantly improves several parameters of male reproductive function in mice, including sperm concentration, viability, motility, morphology, testicular histology, and serum testosterone levels. Phytochemical analysis confirmed that *C. caesia* contains abundant flavonoids, phenolics, tannins, alkaloids, and terpenoids, contributing to its strong antioxidant properties. GC-MS analysis revealed that terpenoid compounds—specifically sesquiterpenes such as β elemene, germacrene, α--humulene, valencene, and curzerene—constitute the majority of the extract. These substances possess well-documented antioxidant, anti-inflammatory, and antibacterial qualities, which likely explain the observed improvements in male reproductive indices (Shabbir and Gashti, 2026; Malel, 2026).

These bioactive metabolites play a critical mechanistic role in scavenging reactive oxygen species (ROS) and maintaining intracellular redox balance. Oxidative stress is a major contributor to male infertility, as excessive ROS can damage sperm membranes, disrupt mitochondrial function, and induce DNA fragmentation in germ cells (Wang et al., 2025; Rocca et al., 2025). Therefore, the high concentration of antioxidant phytochemicals in *C. caesia* likely protects testicular tissues, preserves spermatogenic cell integrity, and supports normal reproductive function.

In this study, treatment with *C. caesia* significantly increased sperm viability and reduced mortality compared with the control group. Improved viability suggests the extract helps maintain cellular metabolic stability and membrane integrity. Mechanistically, antioxidant metabolites may enhance the activity of endogenous antioxidant enzymes—such as superoxide dismutase (SOD), catalase (CAT), and glutathione peroxidase (GPx)—thereby strengthening cellular defense systems. Recent studies have demonstrated that curcumin and related phytochemicals in *Curcuma* species enhance sperm survival and reduce lipid peroxidation in sperm membranes (Lin et al., 2025; Bouhadana et al., 2025).

Conversely, the increase in sperm mortality observed at the highest dose (1,092 mg/kg BW) suggests that excessive concentrations of phytochemicals may exert pro-oxidant or cytotoxic effects. At high levels, these metabolites can disrupt cellular redox homeostasis and promote secondary oxidative stress (Wang et al., 2025).

Sperm motility is a key indicator of male fertility, reflecting the functional capacity of spermatozoa to reach and fertilize the oocyte. In this study, *C. caesia* extract significantly improved motility at moderate doses (364 and 728 mg/kg BW), with the peak observed at 728 mg/kg BW. This improvement is likely attributed to the preservation of mitochondrial function and enhanced ATP production, both of which are essential for flagellar movement. Phenolic metabolites and related antioxidants protect mitochondrial membranes from oxidative damage, sustaining the energy production required for motility (Bouhadana et al., 2025).

Histological examination revealed significant improvements in testicular architecture following extract administration. Doses of 364 and 728 mg/kg BW increased seminiferous tubule diameter, lumen diameter, germinal epithelium thickness, and the number of spermatogenic cells. These structural enhancements indicate improved spermatogenic activity and tissue organization. Active spermatogenesis is reflected in the increased germinal epithelium thickness and the higher density of spermatozoa in the lumen.

Furthermore, *C. caesia* extract influenced endocrine function, as evidenced by increased serum testosterone levels. The highest testosterone concentration was observed in the high-dose group, correlating with increased Leydig cell numbers. While antioxidant-mediated protection of Leydig cells preserves steroidogenic enzyme activity, the highest testosterone levels (in the 1,092 mg/kg BW group) did not correspond to optimal reproductive outcomes. Despite elevated testosterone, sperm quality and histological parameters were superior in the 728 mg/kg BW group. This paradox suggests that high-dose exposure may induce excessive oxidative stress that overrides the beneficial effects of increased testosterone (Sengupta et al., 2024).

However, at the dose (1,092 mg/kg BW), several histological parameters declined, including seminiferous tubule organization and germinal epithelium thickness. The seminiferous tubules appeared less compact, with fewer spermatozoa in the lumen compared with moderate-dose groups. These findings indicate a dose-dependent biphasic (hormetic) response, where beneficial antioxidant effects at moderate doses shift toward pro-oxidant and cytotoxic effects at higher concentrations. Excess phytochemicals may exert prooxidant effects by undergoing autooxidation or interfering with the mitochondrial electron transport chain, leading to increased ROS production (Wang et al., 2025).

In addition to structural effects, *C. caesia* extract influenced endocrine function, as evidenced by increased serum testosterone levels. The highest testosterone concentration was observed in the high-dose group, along with a positive correlation between Leydig cell number and testosterone levels. Leydig cells are the primary site of testosterone synthesis, and their proliferation likely contributes to enhanced androgen production. Antioxidant-mediated protection of Leydig cells may preserve steroidogenic enzyme activity and support testosterone biosynthesis under oxidative stress conditions (Rocca et al., 2025). However, it is important to note that the highest testosterone levels did not correspond to optimal reproductive outcomes. Despite elevated testosterone in the (1,092 mg/kg BW dose group, sperm quality and histological parameters were superior at the dose (728 mg/kg BW), suggesting that balanced hormonal regulation is more beneficial than excessive stimulation.

Recent molecular evidence further supports the role of curcumin and related phytochemicals in regulating testicular function. These metabolites(s) have been shown to modulate protein O-GlcNAcylation homeostasis and restore cellular signaling pathways involved in spermatogenesis (Wang et al., 2025). Additionally, the hypothalamic–pituitary–gonadal (HPG) axis plays a central role in regulating reproductive function through coordinated hormonal signaling. Oxidative stress can disrupt this axis, impairing testosterone production and spermatogenesis, whereas antioxidant metabolites(s) may help maintain its functional integrity (Rocca et al., 2026; Garcia-Lopez et al., 2026).

Although the P3 group exhibited the highest testosterone levels, sperm quality parameters were significantly impaired. This paradox suggests that high-dose exposure may induce excessive oxidative stress within the testicular microenvironment. Oxidative stress, characterized by an imbalance between reactive oxygen species (ROS) and antioxidant defences, is a well-established contributor to male infertility (Kaltsas et al., 2026). Elevated ROS levels can trigger lipid peroxidation of sperm membranes, DNA damage, and mitochondrial dysfunction, ultimately leading to decreased sperm motility, viability, and overall fucntion (Sengupta et al., 2024). Furthermore, spermatozoa are particulary vulnerable to oxidative damage due to their limited antioxidant capacity (Aitken et al., 2026). Therefore, despite increased testosterone levels, the detrimental effects of oxidative stress at higher levels may override the beneficial role of testosterone in supporting spermatogenesis.

Despite these promising findings, several limitations remain. This study utilized a murine model; species-specific differences in metabolism may limit direct extrapolation to humans. Additionally, the underlying molecular mechanisms were inferred from existing literature rather than directly measured via molecular assays. Future research should incorporate biochemical assays to validate specific antioxidant and endocrine pathways.

From a translational perspective, *C. caesia* shows potential as a natural therapeutic agent for managing male infertility associated with oxidative stress. However, careful dose optimization is essential, as excessive intake may lead to adverse pro-oxidant effects. Clinical studies are required to confirm the efficacy, safety, and appropriate dosing strategies in human populations.

## 5 Conclusion

The present study demonstrates that the rhizomes of *C. caesia* contain abundant phytochemicals, including phenolic metabolites, flavonoids, tannins, and alkaloids, which contribute to its significant antioxidant capacity. Using GC-MS analysis, 25 phytochemical substances—primarily from the sesquiterpenoid class—were identified in the *C. caesia* extract. Among these, β-elemene was the primary constituent with the highest relative concentration (24.34%), followed by camphor (10.57%), germacrene B (10.09%), valencene (7.89%), curzerene (6.95%), and $\alpha$-humulene (6.86%).

The administration of *C. caesia* extract improved male reproductive parameters in mice in a dose-dependent manner. Treatments P1 (364 mg/kg BW) and P2 (728 mg/kg BW) significantly enhanced sperm quality, including concentration, viability, morphology, and motility, compared to the control group. These treatments also improved testicular histological structure, as evidenced by increased seminiferous tubule diameter, lumen diameter, and germinal epithelium thickness, alongside higher counts of spermatogenic and Sertoli cells. Among the groups, the medium dose (P2) produced the most pronounced improvements, suggesting it represents the optimal concentration for enhancing spermatogenesis.

In contrast, while the highest dose (P3: 1,092 mg/kg BW) resulted in the greatest increase in Leydig cell numbers and serum testosterone levels, sperm quality and histological parameters were lower than those observed in the P1 and P2 groups. This indicates that excessive doses may exert pro-oxidant effects that disrupt cellular redox balance and impair testicular cell integrity.

Overall, these findings suggest that *C. caesia* ethanol extract enhances male reproductive function by protecting testicular cells and improving spermatogenic activity. This study demonstrates significant improvements in key fertility parameters and identifies both an optimal effective dose and a threshold for potential toxicity—a distinction not clearly established in earlier literature. Furthermore, the GC-MS analysis revealed multiple bioactive metabolites with anti-inflammatory and antimicrobial properties that may underlie these therapeutic effects. These results provide novel preclinical evidence supporting the potential of *Curcuma caesia* in the management of male infertility and establish a robust basis for future clinical investigations.

## Data Availability

The original contributions presented in the study are included in the article/supplementary material, further inquiries can be directed to the corresponding author.
